# Letter to the Editor: “Deepening the mechanistic and clinical translation of PTGDS-driven immunosuppression in esophageal squamous carcinoma”

**DOI:** 10.1097/JS9.0000000000003435

**Published:** 2025-09-05

**Authors:** Qiang Zhang, Gang Tian

**Affiliations:** a Department of Laboratory Medicine, The Fourth Affiliated Hospital of Southwest Medical University, Meishan Sichuan, China; b Department of Laboratory Medicine, The Affiliated Hospital of Southwest Medical University, Sichuan Province Engineering Technology Research Center of Molecular Diagnosis of Clinical Diseases, Molecular Diagnosis of Clinical Diseases Key Laboratory of Luzhou, Sichuan, China

*Dear Editor,*

Zhong *et al*’s study presents a significant investigation into the impact of prostaglandin D_2_ synthase (PTGDS) in esophageal squamous cell carcinoma (ESCC)^[1]^. Their findings reveal that PTGDS deficiency promotes an immunosuppressive tumor microenvironment by inducing M2 macrophage polarization and activating the PI3K/AKT pathway. Utilizing weighted gene co-expression network analysis, bulk transcriptomics data from various cohorts (GSE53624, GSE121931, and the Gaozhou cohorts), immune cell deconvolution, and in vitro and in vivo functional experiments, the authors have identified PTGDS as an independent prognostic indicator. Furthermore, they have developed a nomogram capable of accurately predicting 1-, 3-, and 5-year overall survival rates. The high presence of myeloid-derived suppressor cells, low T cell density, and compromised cytotoxic activity in PTGDS-deficient tumors underscore the intricate immunosuppressive alterations linked to PTGDS depletion. When preparing this letter, we adhered to the TITAN 2025 guidelines on the use of artificial intelligence in academic publishing^[2]^.

Further clarification is required to transition PTGDS from a descriptive biomarker to a therapeutic target. The causal relationship between PTGDS deficiency and AKT hyperactivation remains incompletely understood, with existing data indicating correlation rather than direct causation. To address this gap, inducible PTGDS knockdown models in ESCC cell lines co-cultured with macrophages, coupled with time-course analyses of PI3K/AKT phosphorylation, are necessary to determine if PTGDS loss alone initiates this signaling pathway^[3]^. Subsequent rescue experiments, involving the reintroduction of PTGDS or supplementation of downstream PGD_2_ while concurrently inhibiting related lipid enzymes, are essential to confirm pathway specificity and exclude compensatory effects. Pinpointing a specific intervention point, whether at the level of PTGDS enzymatic activity, PGD_2_ receptor activation, or downstream PI3K/AKT components, would elucidate this mechanistic axis.

Furthermore, linking the nomogram’s prognostic ability to therapeutic response would enhance its clinical usefulness. It is currently unclear whether patients with low PTGDS levels benefit less from checkpoint inhibitors or chemoradiation, despite their association with poorer survival. Analyzing PTGDS expression in ESCC cohorts treated with anti-PD-1/PD-L1 agents or neoadjuvant chemoradiation retrospectively could help determine if PTGDS-low status predicts resistance to treatment. By incorporating PTGDS into the stratification of prospective trials, its role as a companion diagnostic could be validated. For instance, enrolling patients based on PTGDS levels to compare response rates, progression-free survival, and treatment-related side effects could be beneficial. These translational studies could lead to a precision oncology approach where PTGDS measurement guides both risk assessment and personalized therapy selection.

To achieve these objectives, we propose two complementary approaches. Firstly, we will employ CRISPR to disrupt PTGDS in organoids derived from patients and co-culture them with autologous macrophages to simulate tumor-immune interactions in a physiological setting and elucidate the impact of PI3K/AKT modulation^[4]^. Analyzing archived specimens from completed ESCC immunotherapy trials (e.g., KEYNOTE, CheckMate series) for PTGDS IHC or transcript levels and correlating these with objective response and survival metrics will swiftly establish predictive validity^[5]^. Furthermore, integrating PTGDS status with immune-exclusion scores (TIDE, quanTIseq) across extensive cohorts could enhance multi-parameter models that inform immunotherapy strategies beyond PD-L1 expression alone.

Addressing these mechanistic and translational gaps could elevate PTGDS from a promising prognostic marker to an actionable biomarker that guides targeted modulation of the PI3K/AKT axis and enhances immunotherapeutic effectiveness in ESCC. These advancements would not only enhance our molecular comprehension, but also provide concrete clinical advantages for patients battling this aggressive cancer.

## Data Availability

None.
